# Absence of lymphatic vessels in term placenta

**DOI:** 10.1186/s12884-020-03073-w

**Published:** 2020-06-29

**Authors:** Jürgen Becker, Gilles E. Tchagou Tchangou, Sonja Schmidt, Christina Zelent, Fritz Kahl, Jörg Wilting

**Affiliations:** 1 Deparment of Anatomy and Cell Biology, University Medical School Goettingen, UMG, Kreuzbergring 36, 37075 Göttingen, Germany; 2 Department of General-, Visceral- and Pediatric Surgery, University Medical Center Goettingen, UMG, Göttingen, Germany

**Keywords:** Lymphatic endothelial cell - placenta - PROX1 - CCBE1 - PDPN - endometrium - anti-lymphangiogenesis

## Abstract

**Background:**

There has been debate about the existence of lymphatic vessels in placenta. Lymphatic endothelial cell (LEC) markers such as LYVE-1 and podoplanin/D2–40 have been found, although PROX1 has not been detected. The most reliable marker for LECs is the double staining for CD31 and PROX1, which has not been performed yet.

**Methods:**

We studied three term placentas and dissected them into three areas: i.) basal plate area, ii.) intermediate area, and iii.) chorionic plate area. We used immunofluorescence single and double staining with antibodies against CD31, PROX1, LYVE-1, VEGFR-3, D2–40/PDPN, CD34, CCBE-1, and vimentin, as well as nested PCR, qPCR, Western blot and transmission electron microscopy (TEM).

**Results:**

At TEM level we observed structures that have previously mistakenly been interpreted as lymphatics, however, we did not find any CD31/PROX1 double-positive cells in placenta. Absence of PROX1 was also noted by nested PCR, qPCR and Western blot. Also, LEC marker VEGFR-3 was expressed only in a small number of scattered leukocytes but was absent from vessels. The LEC marker D2–40/PDPN was expressed in most stromal cells, and the LEC marker LYVE-1 was found in a considerable number of stromal cells, but not in endothelial cells, which were positive for CD31, CD34, CCBE-1 and vimentin. Additionally, vimentin was found in stromal cells.

**Conclusions:**

Our studies clearly show absence of lymphatics in term placenta. We also show that the functional area of the mother’s endometrium is not penetrated by lymphatics in term pregnancy.

## Background

Placenta and umbilical cord have been studied extensively during the last decades [1–4], and it is well accepted that the umbilical cord at term contains two arteries and one vein. Knowledge about the umbilical vasculature goes back, at least, to Andreas Vesalius (1514–1564) (for literature see: [5]). Lymphatics have never been found in the umbilical cord. However, the existence of lymphatics in placenta cannot be completely excluded, since not all lymphatics finally reach the jugulo-subclavian angle of the venous system. In the eye, Schlemm’s canal, a lymphatic-like vessel, drains into the vortex veins [6]. The heart, too, contains organ-specific lymphatics, which enter a vein at the base of the organ proper [7]. It could therefore not be excluded that the placenta may contain lymphatics, which do not pass through the umbilical cord, but might directly enter placental veins. In fact, at TEM level, there were descriptions of lymphatics in placental villi [8].

The most reliable marker for staining of lymphatic vessels in human tissues is the double-staining with antibodies against the cell adhesion molecule CD31/PECAM-1 and the transcription factor PROX1 [9]. Thereby, CD31 is a pan-endothelial marker for both blood vessels and lymphatics, while PROX1 is found specifically in lymphatic endothelial cells (LECs) in addition to some non-endothelial cell types, which are CD31-negative [10, 11]. There are other highly specific LEC markers, which have frequently been used to identify lymphatics: such as the CD44 homolog LYVE-1 [12], the 43 kDa surface glycoprotein podoplanin (PDPN)/D2–40 [13], 1999), and the Vascular Endothelial Growth Factor Receptor-3 (VEGFR-3 / FLT4) [14–16]. These markers have been used to study human placenta, and thereby, expression of LYVE1 [17] and PDPN/D2–40 has been found [18, 19]. Thereby, PDPN/D2–40 was observed in the placental stroma, and it was suggested that this may be indicative of a reticular-lymphatic-like conductive system. The expression of D2–40 in stromal cells was also found in another study [20]. These authors also noted absence of PROX1 in fetal placental vessels and, correspondingly, absence of lymphatics in placenta. The above cited studies used immunohistochemistry and peroxidase staining with just single primary antibodies. Here we applied double-immunofluorescence techniques, which is necessary to identify LECs with PROX1 and CD31 antibodies. Additionally, we used other recently described LEC markers such as vimentin and CCBE1 [21], and we performed qPCR and nested PCR as highly sensitive methods to study expression of PROX1.

## Methods

### Tissues and cells

Three term placentas (week 38, 40, 41) were collected with the informed, written consent of the mothers after normal vaginal births of healthy children, and dissected into three areas: i.) basal plate area, ii.) intermediate area, and iii.) chorionic plate area. Specimens of app. 1cm^3^ (and predominantly made up of villous tissue) were rinsed in phosphate buffered saline (PBS) and freshly frozen for Western blot analysis and mRNA isolation for PCR, or fixed for histology and immunohistology as described below. Foreskin specimens from healthy boys were used as positive controls for PCR studies and immunohistology of lymphatic vessels as described [21]. The studies were performed with the informed and written consent of the donors or their legal representatives. Human lymphatic endothelial cells (LECs) were purchased from PromoCell (Heidelberg, Germany), cultured in LEC medium and checked for purity as described recently [22]. All studies on human tissues were approved by the ethics committee of the University Medical Hospital Göttingen, UMG (application no. 18/1/18).

### Immunohistology

For immunofluorescence studies, specimens were fixed for 20–25 min in 4% paraformaldehyde (PFA), rinsed in PBS, transferred into 10 and 30% sucrose in PBS, and embedded in tissue freeze medium (Tissue Tek, Sakura Finetek Zoeterwoude, NL). Sections of 12 – 14 μm were incubated with the following primary antibodies: Rabbit-anti-human CCBE1 (1:500, Sigma-Aldrich, München, Germany; #R38605), mouse-anti-human CD31 (1:50, BD Pharmingen; clone WM59), mouse-anti-human D2–40/podoplanin (1:200, Dako, Hamburg, Germany; #M3619), rabbit-anti-human LYVE-1 (1:500, ReliaTech, Wolfenbüttel, Germany; #102-PA50AG), rabbit-anti-human PROX1 (1:500, ReliaTech; #102-PA32AG), mouse-anti-human vimentin (1:200, Dako; #GA630), mouse-anti-human CD34 (1:200, Dako; clone QBEnd-10), mouse-anti-human VEGFR-3 (1:100, ReliaTech; #101-M36). Secondary antibodies were: goat-anti-mouse Alexa 488/594 (#A11001; #A21135), goat-anti-rabbit Alexa 594 (#A11012), donkey-anti-goat Alexa 488 (#A11055; MobiTech, Göttingen, Germany). Sections were counter-stained with Dapi and mounted under cover slips with Fluoromount-G (Southern Biotechnology, US). Photos were taken with AxioImagerZ1 (Zeiss, Göttingen, Germany).

### Transmission electron microscopy (TEM)

Specimens were fixed with original Karnovsky’s fixative over-night [23], washed in 0.15 M phosphate buffer for 10 min, transferred into osmium tetroxide solution and incubated for 2 h at 4 °C. Then the samples were rinsed with 0.15 M phosphate buffer for 10 min and subsequently dehydrated in an ascending series of ethanol. Samples were transferred into Epon embedding solution, cut with an Ultracut E microtome (Reichert-Jung) into 90 nm sections. Staining of sections was performed as described [21]. Specimens were analyzed with Leo 906E TEM (Zeiss, Germany).

### Western blot

Cells and tissues were washed 3-times with DPBS (Pan Biotech; Aidenbach, Germany) and 1 mM sodium orthovanadate (Sigma-Aldrich; Steinheim, Germany), homogenized (tissues), and subsequently lysed with RIPA buffer for 10 min on ice. Samples were transferred to micro-tubes (Sarstedt; Nürnberg, Germany) and centrifuged by 20,800x g for 15 min. Protein lysates were collected and used for SDS-PAGE according to standard procedures. Proteins were transferred to PVDF membranes (Roth; Karlsruhe, Germany), which were then washed with TBST (0.1% Tween 20) for 10 min and blocked for 1 h in blocking buffer (5% BSA in TBST 0.1%). Primary anti-human antibodies were: Lyve1 (ReliaTech, Lot 1408R07), PROX1 (Reliatech Lot 0810R19–1), VEGFR-3/FLT4 (ReliaTech, Lot #9D9) and peroxidase-conjugated α-tubulin, 1:10000 (Abcam, ab40742). Incubation was at 4 °C overnight. After washing with TBST (0.1% Tween 20) for 10 min., the peroxidase-conjugated secondary anti-rabbit IgG, 1:2000 (Santa Cruz; USA) were incubated for 1 h. After washing, antigen-antibody complexes were visualized with ECL in Chemidoc Touch Imaging System (Bio-Rad, München, Germany).

### Real-time RT-PCR

Real Time RT-PCR was performed as described [24]. Primers were:

PROX1_fwd: 5′-tgaatccccaaggttctgag-3′, Prox1_rev: 5′-agcagcttgcggagtacatt-3′, LYVE-1_fwd: 5′-gctttccatccaggtgtcat-3′, Lyve-1_rev: 5′-agcctacaggcctccttagc-3′, Podoplanin_fwd: 5′-gaagacatccccagtcctca-3′, Podoplanin_rev: 5′-ctggatggtgctgagacaga-3′. Relative expression was calculated in comparison to LEC expression levels by using the δδCT-Method [25]. Statistical analyses were conducted by two-way-ANOVA with STATISTICA software (Statsoft, Tulsa, Oklahoma). Normal distribution was verified before testing. The standard error of the mean (SEM) was calculated and is shown in the graphs.

### Nested RT-PCR

Nested PCR was performed to increase specificity and reduce non-specific binding of primers, starting with 40 cycles with outer primers (out) followed by inner primers (in). Primers were:

Prox1_out_fwd: 5′-gtcatctcaccacctgagcc-3′; Prox1_out_rev: 5′-tggaacctcaaagtcatttgct-3′.

Prox1_in_fwd: 5′-gagtgcggcgatcttcaa-3′; Prox1_in_rev: 5′-ggtgacaatccttcctgcat-3′.

## Results

**Fig. 1:**
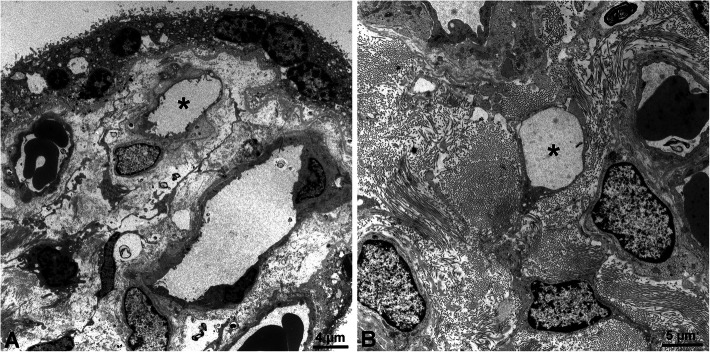
TEM studies of term placentas. **a**, **b**) The chorionic villi are typically made up of syncytiotrophoblast, some cytotrophoblast cells, blood capillaries and stromal cells. Asterisks mark huge vacuolated cells

**Fig. 2:**
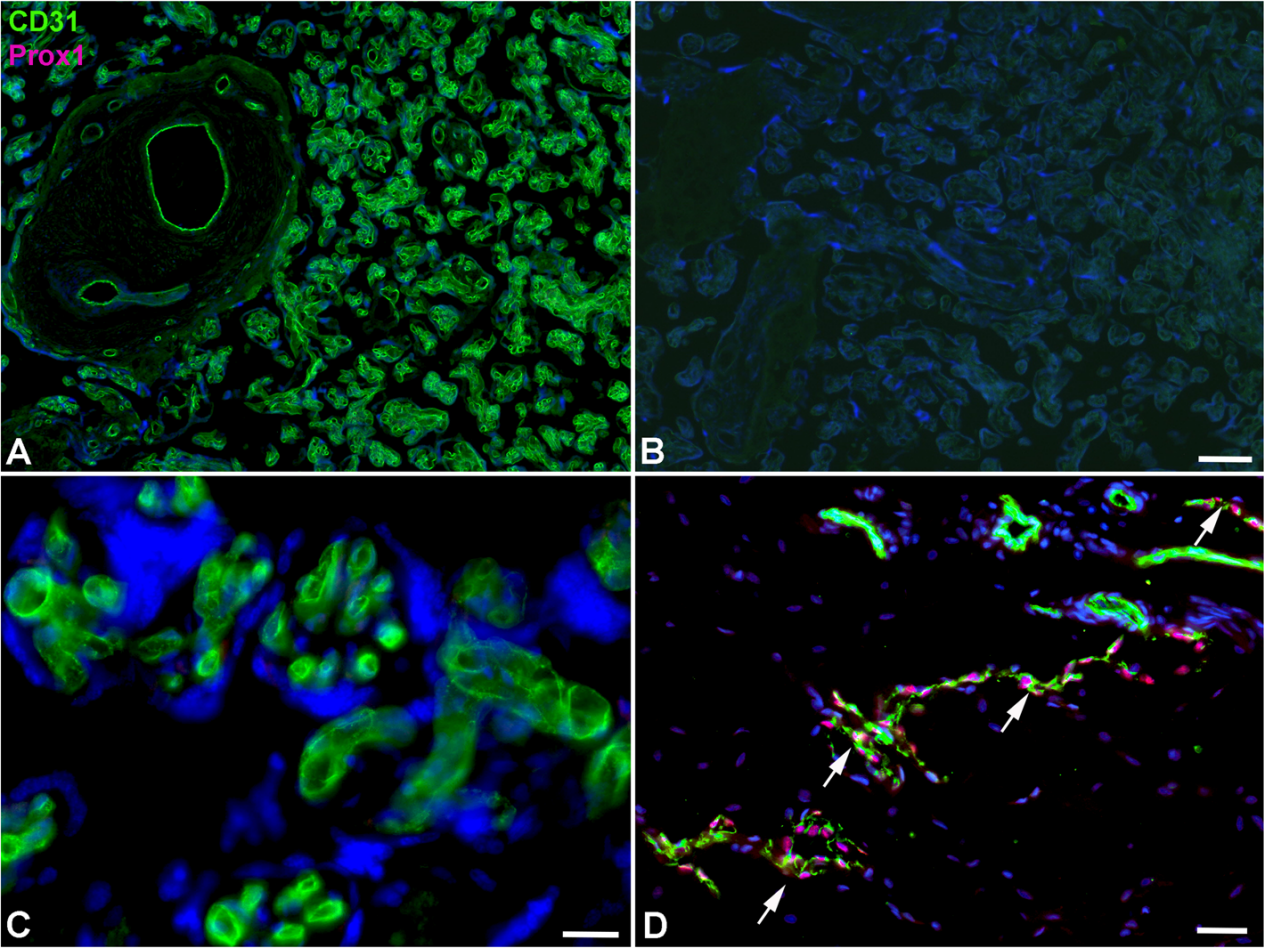
Anti-CD31 (green) and anti-PROX1 (magenta) double-staining of delivered placenta (A-C) and fore-skin (D). **a** CD31 marks all blood vessels in the placenta. **b** Negative control without primary antibody. **c** Higher magnification of A). Note absence of PROX1 in placental vessels. **d** Fore-skin (as positive control) contains a dense network of lymphatics (arrows). Bar = 100 μm in A,B; 15 μm in C, and 35 μm in D

**Fig. 3:**
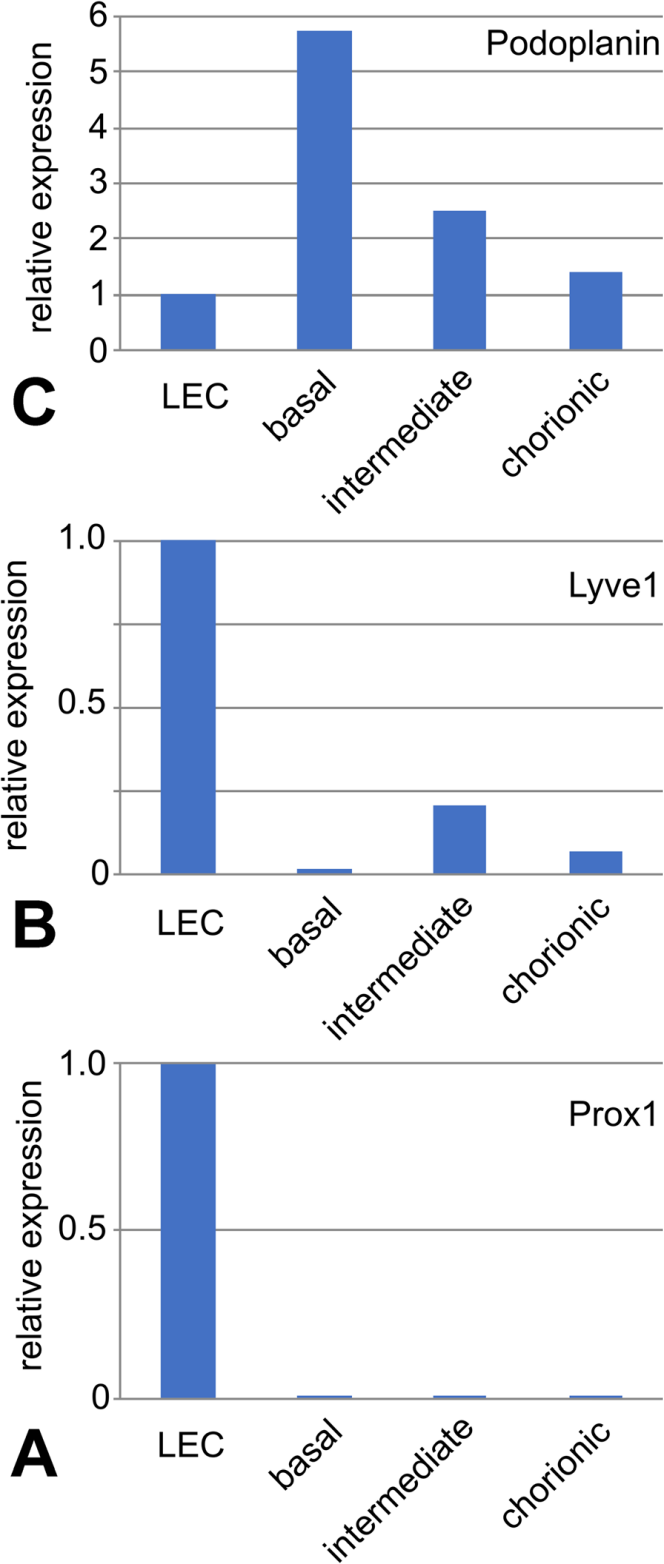
Real-time RT-PCR studies of placenta in comparison to human LECs. Typical example showing expression of **a**) PROX1, **b**) LYVE1 and **c**) podoplanin (PDPN) in a mixture of LECs, and set to 1. PDPN is highly expressed in all areas (basal plate area, intermediate area, chorionic plate area) of the placenta. LYVE1 is also found in all areas but at lower levels. PROX1 is not expressed

**Fig. 4:**
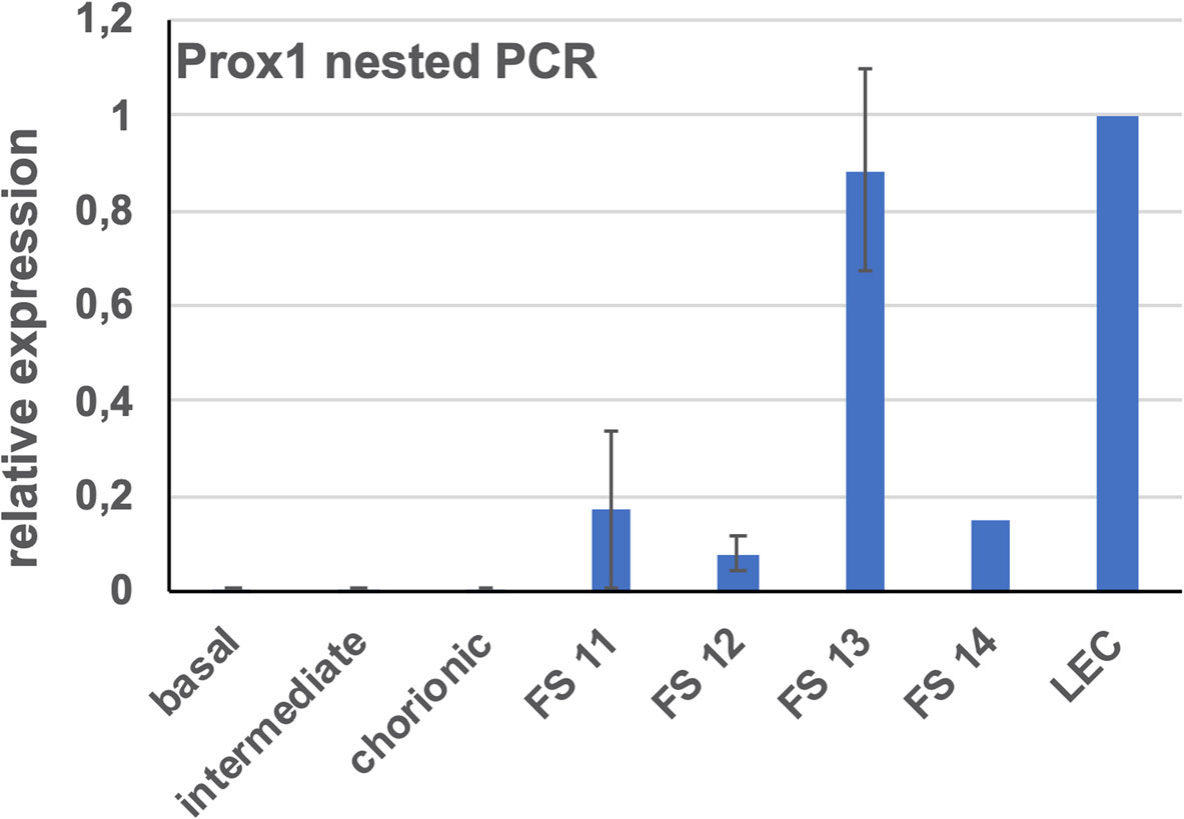
Nested PCR comparing PROX1 expression in placenta, foreskin and LECs. PROX1 expression in a mixture of LECs was set to 1. Note that there is a clear signal in foreskin specimens (FS11–14), while no signal is found in placenta (basal plate area, intermediate area, chorionic plate area)

**Fig. 5:**
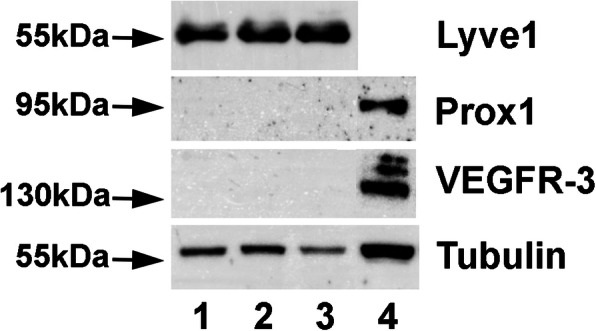
Western blot analysis of placenta (lanes 1–3) in comparison to a mixture of human LECs (lane 4) with antibodies against LYVE1, PROX1, VEGFR-3, and α-tubulin as a housekeeper protein. Lane 1 - basal plate area; lane 2 - intermediate area; lane 3 - chorionic plate area; lane4 - LECs. LYVE1 is expressed in placenta, but PROX1 and VEGFR-3 are not detectable

**Fig. 6:**
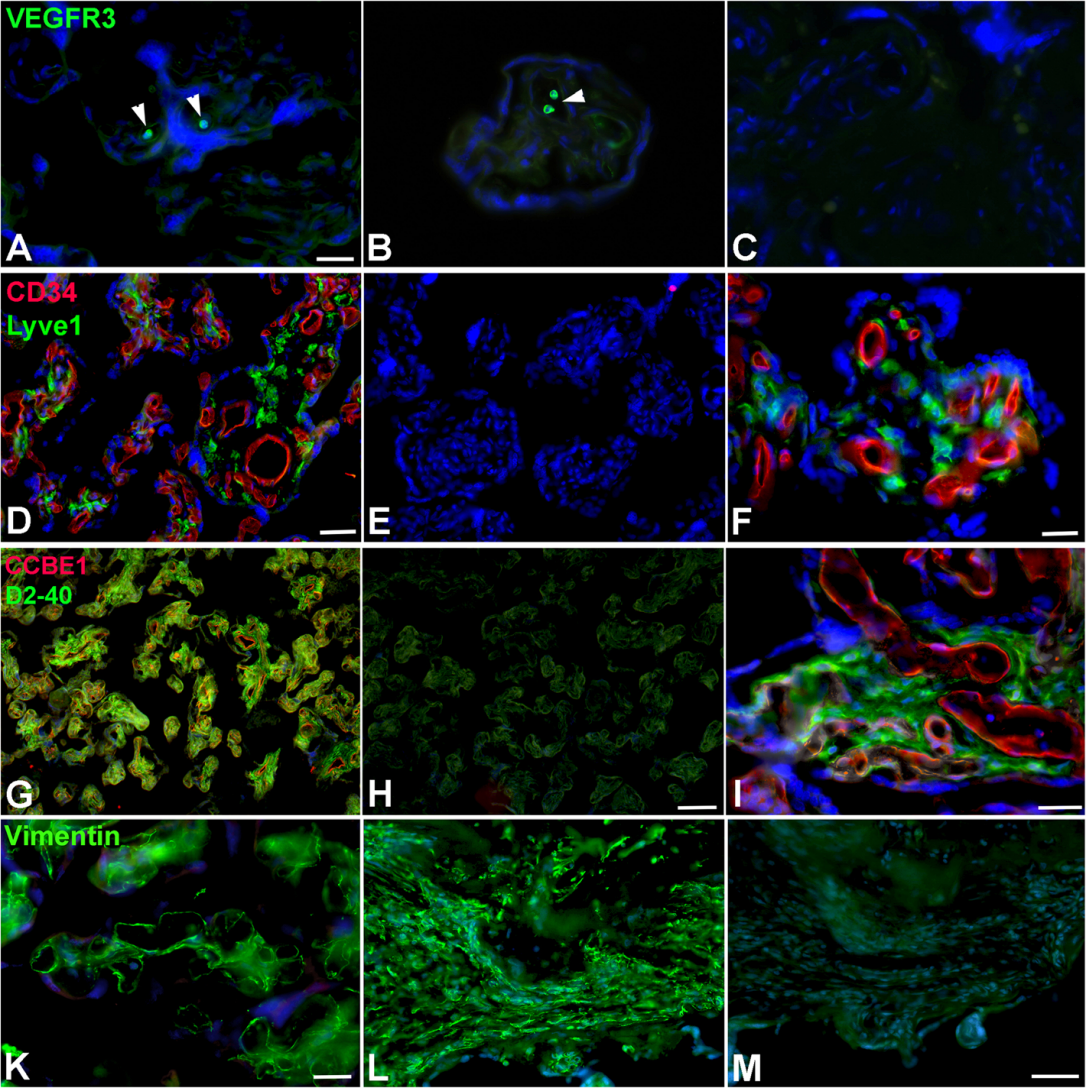
Immunofluorescence staining of term placenta with various antibodies. Counter-staining of nuclei with Dapi (blue). **a**-**c**) VEGFR-3 (green) is expressed in a few intra- and extra-vascular leukocytes (arrowheads in A,B). Bar = 35 μm. C) Negative control without primary antibody. **d**-**f**) CD34 (red) and LYVE1 (green) double-staining. CD34 is expressed in endothelial cells, LYVE1 in scattered stromal cells. E) Negative control without primary antibody. Bar = 120 μm in D, and 30 μm in F. **g**-**i**) CCBE1 (red) and D2–40/PDPN (green) double-staining. CCBE1 is expressed in endothelial cells, PDPN in numerous stromal cells. H) Negative control without primary antibody. Bar = 120 μm in H, and 30 μm in I. **k**-**m**) Vimentin is expressed in endothelial cells and stromal cells of terminal villi (K) and stem villi (L). M) Negative control without primary antibody. Bar = 30 μm in K, and 120 μm in M

Our TEM studies revealed typical characteristics of the term placenta with syncytiotrophoblast, sparse cytotrophoblast, fetal capillaries and stromal cells (Fig. 1), but also cells with huge vacuoles (asterisks in Fig. 1), which have been taken as a hint for the existence of lymphatics [8], but may be signs of disintegration of tissue after delivery of the placenta. There is no cell membrane lining the vacuoles, and there is no basal lamina, which would be characteristic of capillaries. Searching for lymphatics in the placenta we performed anti-CD31 and anti-PROX1 double-staining. In contrast to dermis, which contains a dense lymphatic network, we never observed lymphatics in the placenta (Fig. 2). The absence of PROX1 was confirmed by qPCR (Fig. 3), nested PCR (Fig. 4) and Western blot (Fig. 5)**.** We compared three areas of the placenta (basal plate area, intermediate area, and chorionic plate area) with pure LECs, and with both LECs as well as foreskin tissue. As expected, LECs were clearly positive for PROX1, whereas no signal was found in placenta (Figs. 3, 4, 5). Also, with nested PCR the signal was highest in LECs and a lower signal was found in foreskin. Again, no signal was detectable in placenta from all three selected areas (Fig. 4). Absence of lymphatics in placenta was confirmed by anti-VEGFR-3 staining. We observed a signal in a very small number of scattered leukocytes, but not in endothelial cells (Fig. 6a-c). Accordingly, a signal for VEGFR-3 was not detectable in placenta with Western blot (Fig. 5). The LEC marker LYVE1 was found in scattered stromal cells of the placenta, and there was no colocalization with the endothelial marker CD34 (Fig. 6 d-f). LYVE1 was well detectable in placenta with qPCR and Western blot (Figs. 3, 5). The LEC marker D2–40/PDPN was detected in almost all stromal cells of the placenta, and again, there was no colocalization with the endothelially expressed CCBE1 (Fig. 6 g-i). With qPCR we found high levels of PDPN expression in placenta (Fig. 3). Vimentin, a typical mesenchymal marker was found in endothelial cells and stromal cells (Fig. 6 k-m). Our data reveal that none of the characteristic LEC markers (PROX1, VEGFR-3, LYVE1, and PDPN) is expressed in endothelial cells of the placenta; and PROX1, the probably most important marker, is completely absent from placenta.

## Discussion

### Lymphatics in placenta

The discussion about the existence of lymphatics in the placenta was refueled by the description of reticular lymphatic-like D2–40/PDPN-positive networks [19]. Previously, at TEM level, vessel-like structures had been described that appeared to be lymphatics [8]. At TEM level, however, lymphatics can only be identified by the characteristic overlapping junctions between LECs, which represent valves, and are associated with anchoring filaments that connect the outer valvular leaflet with adjoining collagen fibers or fibrocytes [26]. We did not detect such ultrastructural characteristics and suggest that the lymphatics described previously in the placenta [8] may represent signs of massive intracellular fluid accumulation. In our eyes, the immuno-histological studies on PDPN shown by Bellini et al. 2012 [19] are not convincing. Nevertheless, PDPN expression in placenta has also been shown by others [18, 20]. Again, these studies used single immunohistochemical staining of placentas, not double immunofluorescence of LEC makers. We therefore reinvestigated this topic. We used well-known and newly identified BEC and LEC markers. We show that placental endothelial cells are positive for CD31, CD34, CCBE-1 and vimentin. There is no co-expression of PROX1 in CD31^+^ vessels, no co-expression of LYVE1 in CD34^+^ vessels, and no co-expression of D2–40 in CCBE1^+^ vessels. These data, together with the non-detectable PROX1 PCR signal, clearly show the absence of lymphatics in placenta.

We show that LYVE1 is expressed in a distinct subpopulation of stromal cells. These cells may, at least in part, represent macrophages, as shown in embryonic and adult tissues [27–29]. D2–40 is expressed in the majority of villous stromal cells, arguing against a specific reticular lymphatic-like system in term placenta. The relation between the intercellular (pre-lymphatic) spaces and the lymphatic vascular system was seminally studied by Friedrich Daniel von Recklinghausen (1862) [30]. Generally, we recommend that the term lymphatic vascular system (lymphatics) should be restricted to a system lined by LECs. This also applies to the para-vascular (Virchow Robin) spaces in the central nervous system, which have been designated glymphatics [31]. In sum, our data confirm the findings by Castro et al. (2011) [20] on the non-existence of lymphatics in term placenta, extend previous studies on the expression of vimentin and CCBE1 in endothelial cells [21], and challenge the existence of a reticular lymphatic-like system in the stroma of term placenta [19]. However, studies on first and second trimester placenta seem to be useful to obtain a more complete view of lymphatic functions in this rapidly changing organ.

### Lymphatics in endometrium

We collected term placentas and dissected them into three areas: i.) basal plate area, ii.) intermediate area, and iii.) chorionic plate area. We expected that the basal plate area might differ from the others with respect to the expression of LEC markers, especially PROX1. However, even with nested PCR, we did not observe significant differences. The basal plate area contains (a small portion of) functional maternal tissue, and it has been shown that the endometrial stroma is invaded by lymphatics (for review see: [32]). It has been estimated that 13 and 43% of vessels in the functional and basal layers of the endometrium, respectively, are lymphatics [33]. Thereby, close association of lymphatics with spiral arterioles was observed. Interestingly, anti-lymphangiogenesis seems to occur during decidualization. PDPN-positive lymphatics have been found in non-decidualized hypersecretory endometrium, in clear contrast to the more superficial decidualized areas [34]. Our data seem to be in line with these findings. The decidua accomplishes roles, among others, in maternal tolerance. In mice, endometrial dendritic cells become trapped during decidualization and lose their potency to migrate into uterine lymphatics and lymph nodes [35]. Anti-lymphangiogenesis in decidualized human endometrium may act in the same functional direction, and may thereby support immune tolerance of the fetal allograft. Rodent endometrium, however, does not contain lymphatics [32], limiting functional studies on this system. In the human, interactions of trophoblast cells with lymphatics have been observed in the first trimester of pregnancy [36]. There, extravillous trophoblast cells invade the wall of arteries and veins to almost the same high extent, while lymphatics are invaded less often. Intraluminal trophoblast cells are found in maternal veins and lymphatics, and, with the latter, may reach the regional lymph nodes. The functional significance of these interactions remains to be studied, however, in recurrent spontaneous abortion the number of trophoblast-affected lymphatics is significantly reduced [36]. The invasion of trophoblast cells into lymphatics takes place even before the trophoblast cells remodel the spiral arteries in the decidua [37], which seems to be in line with the hypothesis that immunomodulation is an early aspect of pregnancy, while later endometrial lymphatics may lose their functional significance.

## Conclusions

Our studies provide clear evidence for the absence of lymphatics in term placenta. Although some LEC markers are highly expressed in placenta, none of these markers is colocalized to endothelial cells. The transcription factor PROX1, which is essential for the development and maintenance of lymphatics, is not expressed in term placenta, neither at protein nor at RNA level. The placenta obviously possesses other mechanisms to handle typical homeostatic functions of the lymphatic vasculature. We could also not detect PROX1 expression in endometrial tissue associated with the placenta specimens. While in the first trimester the endometrial lymphatics seem to have important functions for the establishment of mother’s immunosuppressive mechanisms [36, 37], the importance of lymphatics may become reduced during maturation. If this is accompanied by anti-lymphangiogenesis in decidualized endometrium must be tested comparatively on a larger number of samples.

## Supplementary information

**Additional file 1.**

## Data Availability

All data generated or analyzed during this study are included in this published article and the supplementary information files. Original qPCR data can be sent upon request.

## Abbreviations

BEC: Blood vascular endothelial cell
CCBE1: Collagen and calcium binding EGF domains 1
CD31/PECAM-1: Platelet endothelial adhesion molecule-1
CD34: CD34 molecule
EGM-2: Endothelial growth medium-2
LEC: Lymphatic endothelial cell
LYVE-1: Lymphatic vessel endothelial hyaluronic acid receptor-1
PBS: Phosphate-buffered saline
PDPN: Podoplanin (D2–40)
PROX1: Prospero-related homeobox transcription factor-1
TEM: Transmission electron microscope/microscopy
VEGF-C: Vascular endothelial growth factor-C
VEGFR-3: Vascular endothelial growth factor receptor-3 / FLT4
