# Supplementary figures and images for: Absence of lymphatic vessels in term placenta
Source: BMC Pregnancy Childbirth. 2020 Jun 29;20:380. doi: 10.1186/s12884-020-03073-w (PMC7325062; doi:10.1186/s12884-020-03073-w)

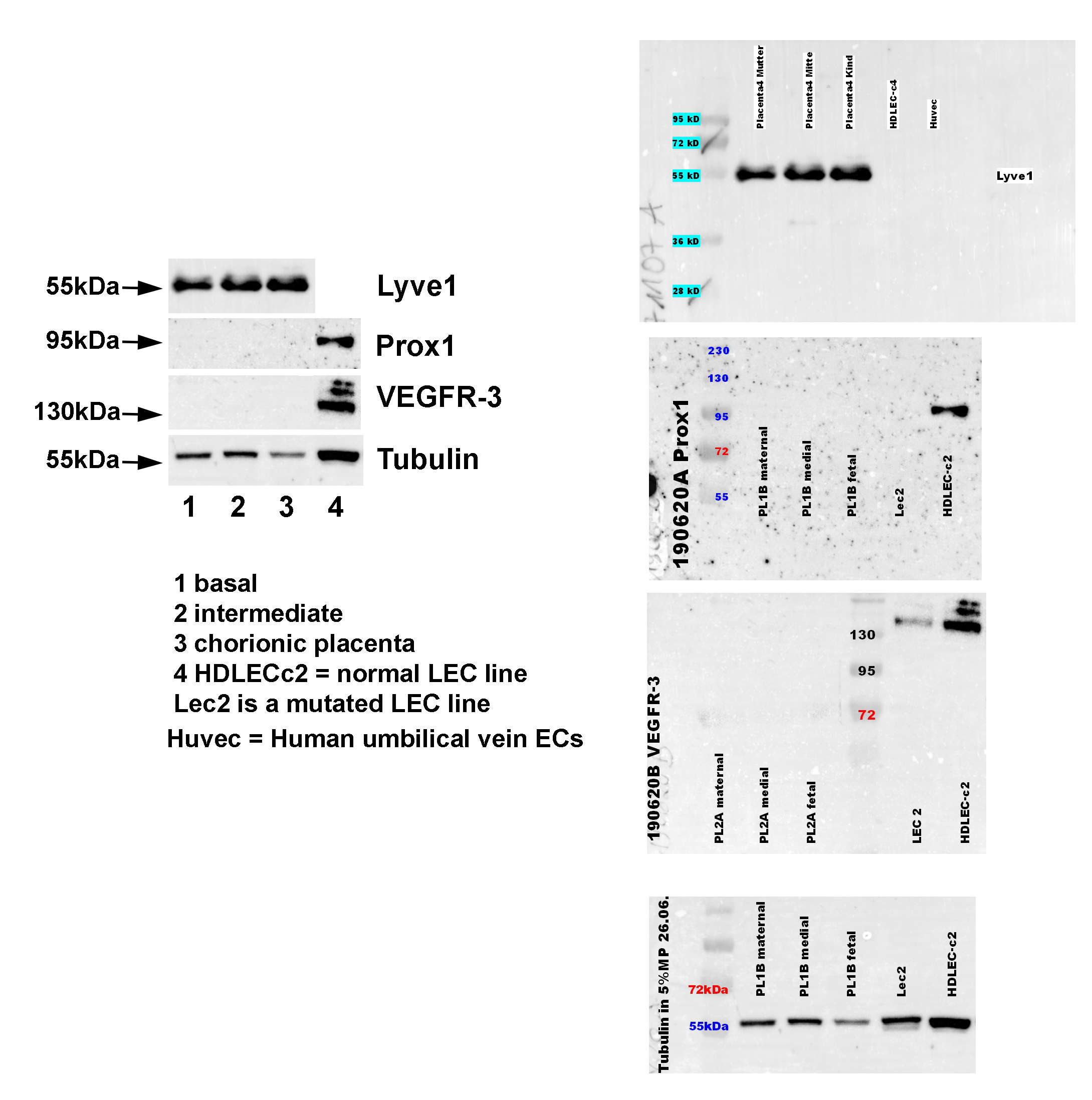

Supplement: Supplementary file 1 — Additional file 1. [file 12884_2020_3073_MOESM1_ESM.jpg]
